# Long-Term Effects of Ionizing Radiation on the Hippocampus: Linking Effects of the Sonic Hedgehog Pathway Activation with Radiation Response

**DOI:** 10.3390/ijms222212605

**Published:** 2021-11-22

**Authors:** Francesca Antonelli, Arianna Casciati, Montserrat Belles, Noemi Serra, Maria Victoria Linares-Vidal, Carmela Marino, Mariateresa Mancuso, Simonetta Pazzaglia

**Affiliations:** 1 Division of Health Protection Technologies, Italian National Agency for New Technologies, Energy and Sustainable Economic Development (ENEA), 00123 Rome, Italy; arianna.casciati@enea.it (A.C.); carmela.marino@enea.it (C.M.); mariateresa.mancuso@enea.it (M.M.); 2 Physiology Unit, School of Medicine, Rovira I Virgili University (URV), 43007 Reus, Spain; montserrat.belles@urv.cat (M.B.); noemi.serra@urv.cat (N.S.); mvictoria.linares@urv.cat (M.V.L.-V.)

**Keywords:** ionizing radiation, astrocytes, hippocampal neurogenesis, cognitive effects

## Abstract

Radiation therapy represents one of the primary treatment modalities for primary and metastatic brain tumors. Although recent advances in radiation techniques, that allow the delivery of higher radiation doses to the target volume, reduce the toxicity to normal tissues, long-term neurocognitive decline is still a detrimental factor significantly affecting quality of life, particularly in pediatric patients. This imposes the need for the development of prevention strategies. Based on recent evidence, showing that manipulation of the Shh pathway carries therapeutic potential for brain repair and functional recovery after injury, here we evaluate how radiation-induced hippocampal alterations are modulated by the constitutive activation of the Shh signaling pathway in Patched 1 heterozygous mice (*Ptch1*^+/−^). Our results show, for the first time, an overall protective effect of constitutive Shh pathway activation on hippocampal radiation injury. This activation, through modulation of the proneural gene network, leads to a long-term reduction of hippocampal deficits in the stem cell and new neuron compartments and to the mitigation of radio-induced astrogliosis, despite some behavioral alterations still being detected in *Ptch1*^+/−^ mice. A better understanding of the pathogenic mechanisms responsible for the neural decline following irradiation is essential for identifying prevention measures to contain the harmful consequences of irradiation. Our data have important translational implications as they suggest a role for Shh pathway manipulation to provide the therapeutic possibility of improving brain repair and functional recovery after radio-induced injury.

## 1. Introduction

Radiation therapy has long been an indispensable tool in the treatment for primary and secondary brain tumors, providing long-term survival benefits for patients. In the United States alone, radiation therapy is used to treat more than 200,000 patients a year, thus representing one of the main treatments for brain cancer. While effectively eliminating cancer cells, irradiation also induces damage in the healthy tissues, leading to severe cognitive impairments in up to 90% of patients undergoing whole-brain radiation therapy [1,2,3,4]. The dentate gyrus (DG) of the hippocampus represents one of the main brain sites of adult neurogenesis. It has been proposed that radiation injury to the neural stem cell compartment of the hippocampus may be involved in the cognitive decline, as well as other hippocampus-related side effects such as emotional alterations, observed during the follow-up of treated patients, particularly children [5,6]. 

Neurogenesis is the process by which new neurons arise from neural stem and progenitor cells, mature, specialize and become integrated and functional within the neuronal network. Although originally believed to occur only during developmental stages, it was demonstrated to occur in adult birds [7], rodents [8,9], and even humans [10,11]. The subgranular zone (SGZ) of the hippocampus contains the neurogenic “niche”, a specific microenvironment permissive for neuronal development that comprises the precursor cells, their immediate progeny and immature neurons, immune cells such as microglia and macrophages, endothelia and the extracellular matrix. Neurogenesis in adults does not occur at a constant rate and is affected by endogenous and exogenous factors, including hormones [12], physical and psychosocial stress [13], physical activity [14], diet [15], enriched environments [14] and age [16]. 

Impairment of neurogenesis in the hippocampus following whole-brain irradiation is now referred to as one of the most important mechanisms of radiation-induced cognitive dysfunction [17,18,19,20]. The critical role of the hippocampus in the pathogenesis of radiation-induced neurocognitive effects is also underlined by the observation that hippocampal avoidance seems to reduce the short-term memory decline in adults receiving whole-brain radiation therapy [21].

The Sonic hedgehog (Shh) signaling pathway is crucial for normal embryonic growth, development and organogenesis of almost all the organs in mammals, as well as in regeneration and homeostasis [22]. The Shh pathway consists mainly of SHH protein, the transmembrane receptor Patched (PTCH1), Smoothened (SMO), and the downstream zinc finger protein (GLI1) transcription factor. In the presence of SHH, PTCH1 relieves its suppression on SMO to activate the downstream transcription factor GLI1 and several downstream pathways. 

Shh signaling has a pivotal role in the formation of neural germinal niches located in the subventricular zone (SVZ) of the lateral ventricles and the subgranular zone of the hippocampal DG, and in nervous system cell type specification [23,24]. Direct proofs of its involvement in establishing neurogenic niches come from mouse studies in which *Shh* or *Smo* were ablated, causing several brain abnormalities including a reduced number of progenitors in the subventricular zone and subgranular zone [25]. Moreover, genetic ablation of the primary cilium, essential for Shh signaling, results in decreased *Shh* target gene expression and a phenotype similar to that of *Smo*-deficient mice [26,27]. In contrast, the constitutive expression of *Smo* (*SmoM2*-*YFP*) results in a marked expansion of the DG [27]. 

SHH has also been implicated in a wide array of events contributing to the maintenance of the adult central nervous system (CNS). We have recently shown that Shh signaling deregulation in mice with germline inactivation of one copy of the *Ptch1* gene (*Ptch1*^+/−^) [28], besides DG patterning defects detectable as elongation and thinning of the blades, also causes deregulation at multiple steps during neurogenesis. In the DG of *Ptch1*^+/−^ adult mice, this is detectable as a reduction of quiescent stem cells and newborn neurons and an accumulation of proliferating intermediate progenitors, indicative of defects in the dynamic transition among neural stages [29]. 

The deregulation of the Shh signaling pathway is important in brain tumor formation, regulation of cancer stem cell proliferation and in the control of tumor invasiveness [30,31,32]. More recently its role in many neurological and mood disorders, such as autism, depression, Parkinson’s disease, Huntington disease, epilepsy, diabetic neuropathy, demyelinating disease, locomotor deficit, cognitive dysfunction, ischemic and traumatic brain injury, has become clear [33,34]. Many evidences have also demonstrated the role of the Shh signaling pathway in CNS repair and regeneration after brain injury. Neuroprotective effects of the Shh signaling pathway in ischemic stroke, in the setting of complex neonatal cerebellar injury and in demyelinated lesions have been reported in recent studies [35,36,37], suggesting important therapeutic possibilities for the modulation of this pathway after brain injury.

In this study we investigated the in vivo long-term consequences of Shh signaling pathway activation on radio-induced effects in the hippocampus to seek for evidence of protection against radiation injury. Interestingly, our results show that constitutive activation of the Shh pathway in *Ptch1*^+/−^ mice mitigates radiation-induced astrogliosis and deficits of neurogenesis, by preserving radiosensitive populations of neural stem and progenitor cells as well as newborn neurons in the SGZ of the DG from detrimental radiation effects. Elucidation of the mechanisms controlled by Shh signaling in the DG in response to radiation may lead to a better understanding of the etiology and treatment of brain radiation injury.

## 2. Results

### 2.1. Constitutive Activation of the Shh Signaling Pathway Mitigates Radiation-Induced Inhibition of Neurogenesis in the Hippocampus

In a previous work we have shown that constitutive Shh pathway activation induces alterations in the dynamic transition among neural stages, accumulating proliferating intermediate progenitors and newborn neurons in the DG of *Ptch1*^+/−^ mice [29]. We therefore investigated the possible effects of Shh pathway activation on the mitigation of radiation-induced deficits in hippocampal neurogenesis, comparing the DG neurogenesis-rate in irradiated wild type (WT) and *Ptch1*^+/−^ mice. To address this, we have examined the radiation-induced modifications in the cellular composition of the subgranular zone of the DG according to criteria based on morphological cellular features and labelling of stage-specific adult neurogenesis markers by immunohistochemical analyses.

**Figure 1:**
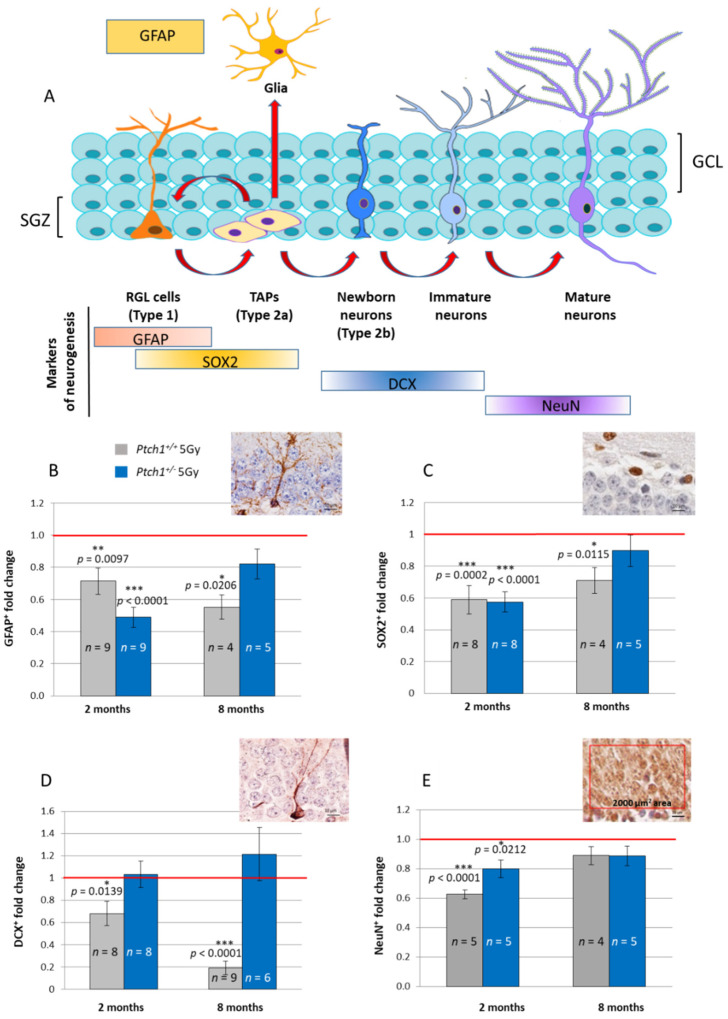
Effect of irradiation with 5 Gy of X-rays at P10 on the lineage-specific composition of DG in WT and *Ptch1*^+/−^ mice at 2 or 8 months post-irradiation. (**A**) Schematic representation of adult neurogenesis in the hippocampal DG and relative stage-specific markers. Representative immunostaining images and quantification for (**B**) glial fibrillary acidic protein (GFAP), (**C**) sex determining region Y (SRY) box 2 (SOX2), (**D**) doublecortin (DCX) and (**E**) mature neurons (NeuN). The red rectangular field of 2000 µm^2^ in E delineates a representative area in which NeuN analysis was carried out. Images, 100× magnification, scale bar = 10 µm. Immunohistochemical analysis for NeuN was carried out in a rectangular field of 2000 µm^2^ in the supra- and infrapyramidal blade. The number of mice used per test is indicated with *n* in the graphs (n). Data are reported as mean ± SEM * p < 0.05; ** p < 0.01; *** p < 0.001 for comparison with controls (Student *t*-test).

As shown in Figure 1A, slowly dividing/quiescent radial glia-like cells (RGLs, type 1 cells), are featured with a single radial process that extends through the granular cell layer of the DG, and express GFAP. RGLs give rise to transit amplifying neural stem cells (TAPs) that are small round cells, expressing a sex determining region Y (SRY) box 2 (SOX2), that enlarge the pool of neurogenic cells (type 2a). The first indications of neuronal lineage choice appear at the level of type 2a cells and involve the suppression of *Sox2* expression, critical for the transition from stem/progenitor cells to newborn neurons labelled by DCX (type 2b cells). Subsequently, newborn neurons progress into mature granule neurons that finally integrate in the existing neuronal circuit.

A comparison of stage-specific cellular markers in WT mice irradiated at P10 with 5 Gy of X-rays vs. unirradiated WT mice, indicates perturbations of adult hippocampal neurogenesis at 2 months after irradiation, detectable as a significant depletion of type1 RGL cells (−29% in GFAP+, *p* = 0.0097; Figure 1B), type 2a cells (−41% in SOX2+, *p* = 0.0002; Figure 1C), newly generated type 2b neurons (−32% in DCX+, *p* = 0.0139; Figure 1D) and the mature neuron density (−37% in NeuN, *p* < 0.0001; Figure 1E). Nearly all the depletions of irradiated WT mice were also detected in irradiated *Ptch1*^+/−^ mice (−51% in GFAP, *p* < 0.0001; −43% in SOX2, *p* < 0.0001; −20% in NeuN, *p* = 0.0212) with the exception of newborn neurons labelled by DCX that were unchanged compared to the control (Figure 1B–E). 

At 8 months after exposure, in WT mice we still observed the significant depletion of almost all the cellular compartments impaired at 2 months post-irradiation, including GFAP+ radial stem cells, SOX2+ neural progenitors and DCX+ newborn neurons (−45% in GFAP, *p* = 0.0206; −29% in SOX2, *p* = 0.0115; and −80% in DCX, *p* < 0.0001), except mature neurons labelled by NeuN that were back to basal levels (Figure 1B–E). These data indicated the long-term consequences of radiation injury on neurogenesis in WT mice, involving quiescent, dividing and differentiating neuronal populations. Notably, in marked contrast, the hippocampi of irradiated *Ptch1*^+/−^ mice showed a complete recovery of all the stage-specific populations at 8 months post-irradiation, returning to the basal levels of the age-matched unirradiated *Ptch1*^+/−^ mice, indicating that neurogenesis was completely restored.

Importantly, our data indicate that the constitutive activation of the Shh pathway mitigates the long-term consequences of irradiation on hippocampal neurogenesis, protecting both staminal and differentiated neuronal populations of the DG from the detrimental radiation effects that in WT, but not *Ptch1*^+/−^ mice, were still detectable as a robust depletion at 8 months post-irradiation. 

### 2.2. Constitutive Shh Pathway Activation Protects the Hippocampus from Radiation-Induced Astrogliosis

Astrocytes represent a cell population with distinctive morphological and functional characteristics and with a regulatory role in brain functions including neurogenesis and synaptogenesis, blood–brain barrier permeability and extracellular homeostasis, that undergo complex region-specific and age-dependent remodeling [27]. 

**Figure 2:**
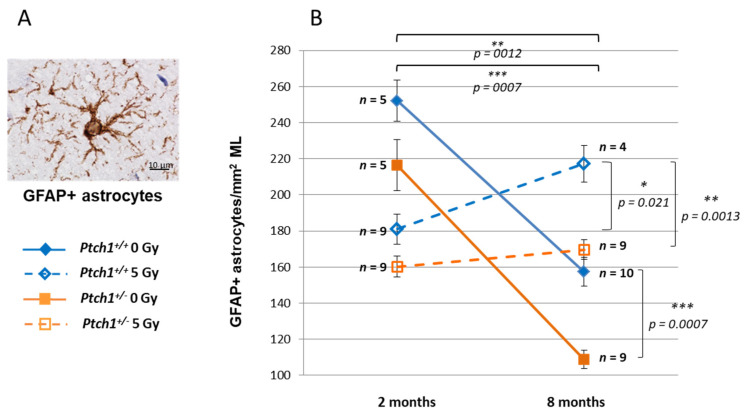
Long-term effect of irradiation with 5 Gy of X-rays at P10 in astrocytes in WT and *Ptch1*^+/−^ mice at 2 or 8 months post-irradiation. (**A**) Representative immunostaining image of GFAP+ astrocytes in the molecular layer (ML) of the hippocampus. (**B**) Astrocyte quantification in unirradiated and irradiated WT and *Ptch1*^+/−^ mice. Image, 100× magnification, scale bar = 10 µm. The number of mice used per test is indicated with *n* in the graphs. Data are reported as mean ± SEM * p < 0.05; ** p < 0.01; *** p < 0.001 for comparison with controls (Student *t*-test).

In this work GFAP, a well-established astrocyte marker, was used to follow the hippocampal astrocyte population in ML over time (Figure 2) [38]. In unirradiated brain labelled with anti-GFAP antibody, we observed a progressive decline in the astrocyte number between 2 and 8 months of age in hippocampi from both WT (−38%, *p* = 0.0012) and *Ptch1*^+/−^ (−49%, *p* = 0.0007) mice, irrespective of the genotype. In agreement with our previously published results [29], we show here that *Ptch1*^+/−^ mice consistently show a lower number of astrocytes compared to WT mice, both at 2 (−16%, *p* = 0.095) and 8 months (−31%, *p* = 0.0007), suggesting a role for the Shh pathway in the control of the astrocyte number. Astrocytes respond to various forms of CNS insults, including ionizing radiation, through a process called reactive astrogliosis, which has become a pathological hallmark of CNS structural lesions [39]. Accordingly, we show that while irradiation initially induces a depletion of astrocytes at 2 months post-irradiation in both genotypes compared to the unirradiated control (−28%, *p* = 0.0063 for WT; −24%, *p* = 0.0161 for *Ptch1*^+/−^), the number of astrocytes tends to increase with time at 8 months post-irradiation. However, while in WT mice we detected a significant increase of 24% in the number of astrocytes at 8 months post-irradiation, compared with 2 months (*p* = 0.021), in *Ptch1*^+/−^ mice the small increase of 6% (*p* = 0.22) was not significant. Moreover, at 8 months post-irradiation WT mice showed a significantly higher number of astrocytes compared to age-matched irradiated *Ptch1*^+/−^ mice (+31%, *p* = 0.0013; Figure 2).

Taken together our results indicate that constitutive Shh pathway activation effectively prevents the progressive radiation-induced astrogliosis, that was is in fact only observed in WT but not in *Ptch1*^+/−^ mice, suggesting a role for Shh signaling in the regulation of the astrocyte population following injury.

### 2.3. Long-Term Effect of Constitutive Shh Pathway Activation on the Expression of Neurogenesis-Related Genes after Irradiation

**Figure 3:**
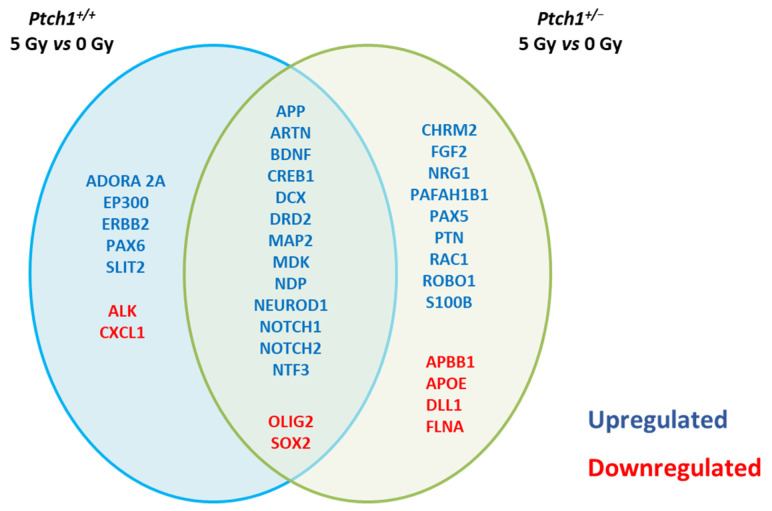
Long-term effect of irradiation with 5 Gy of X-rays at P10 on the expression of neurogenesis-related genes in the hippocampus of WT and *Ptch1*^+/−^ mice at 8 months post-irradiation. Venn diagram of the exclusively deregulated and shared genes in the hippocampus of WT and *Ptch1*^+/−^ mice irradiated vs. their respective unirradiated counterparts. A *p*-value ≤ 0.05 and a fold-regulation > 2 was defined as upregulation (blue); a *p*-value ≤0.05 and fold-regulation of <2 as downregulation (red).

Transcriptional control of adult neurogenesis represents the backbone of neuronal development. To investigate the molecular mechanisms of radiation-induced neurogenesis deficits and how the activation of the Shh pathway restrains this impairment, we evaluated the expression of neurogenesis-related genes in the irradiated hippocampi of WT and *Ptch1*^+/−^ mice. We analyzed and compared RNA expression profiles of 84 genes with established roles in neurogenesis, at 8 months post-irradiation, when the protective effect of Shh pathway activation on radiation-dependent neurogenesis impairment was prominent. To address this, RNA extracted from the micro-dissected DG from unirradiated and 5 Gy irradiated *Ptch1*^+/−^ and WT mice brains, was analyzed through a pathway-based PCR expression array approach. Our analysis showed that about 26% (22/84) and 33% (28/84) of the genes were still significantly deregulated (fold-regulation > 2; *p* ≤ 0.05) in irradiated compared to unexposed WT and *Ptch1*^+/−^ mice, respectively. Most of the deregulated genes (15/22 in WT mice and 15/28 in *Ptch1*^+/−^ mice) were common between irradiated WT and *Ptch1*^+/−^ mice (Figure 3). It is interesting to note that by grouping the deregulated genes into functional categories (Table 1), most of the common deregulated genes fall in the “Synaptic functions” (7/15) and “Cell differentiation” (6/15) groups. This holds true for the exclusively deregulated genes in irradiated *Ptch1*^+/−^ mice, also mainly belonging to the “Synaptic functions” (6/13) and “Cell differentiation” (3/13) groups. Some of the other exclusive genes for *Ptch1*^+/−^ mice fall in the functional group of “Growth factors” (3/13) and “Cell Adhesion/Neural Migration” (3/13). Notably, three of the seven genes exclusively deregulated in irradiated WT mice, belong to the category of “Apoptosis”, representing the most numerous cluster in the exclusive genes for WT mice.

### 2.4. Effect of Constitutive Shh Pathway Activation on Radiation-Induced Changes in Mouse Behaviour

The hippocampus is an area of the brain with functions that are commonly linked with memory or dementia when dysfunctional. However, data suggest that it may also yield important clues about a range of mental health illnesses including addiction, anxiety and depression [40].

**Figure 4:**
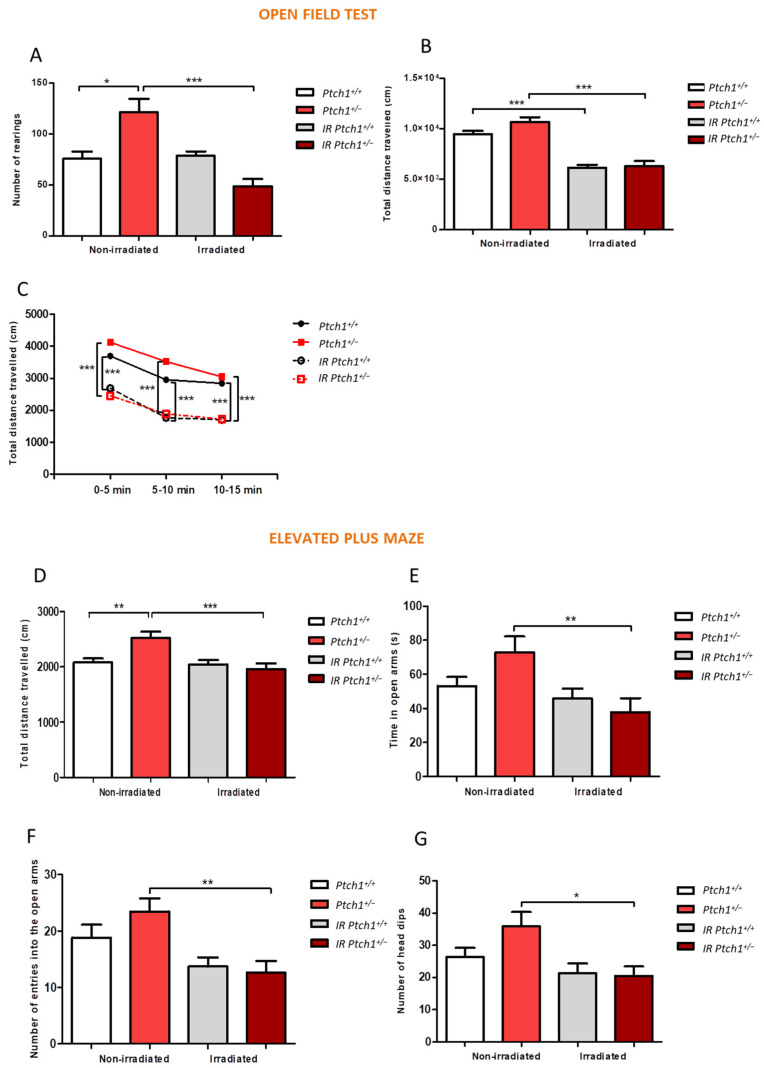
Long-term behavioral effect of irradiation with 5 Gy of X-rays at P10 in WT and *Ptch1^+/^*^−^ mice at 8 months post-irradiation assessed through Open Field (OF) and Elevated Plus-Maze (EPM) tests. WT (*n* = 18) and *Ptch1*^+/−^ (*n* = 15) mice irradiated or left unirradiated (*n* = 17 for WT; *n* = 12 for *Ptch1*^+/−^) were submitted to the tests. (**A**) Number of rearings in OF; (**B**) Total distance travelled in OF; (**C**) Total distance travelled (habituation) in OF. (**D**) Total distance travelled in EPM; (**E**) Time in open arms in EPM; (**F**) Number of entries into the open arms in EPM; (**G**) Number of head dips in EPM. Data are given as the mean ± SEM. * *p* < 0.05; ** p < 0.01; *** p < 0.001. Statistical significance for all tests (described in the “Cognitive Statistical Analysis” paragraph) was established at *p* < 0.05.

To evaluate whether Shh activation modifies the mouse behavioral response to irradiation, we compared the behavior of WT and *Ptch1*^+/−^ mice 8 months post-irradiation through Open Field (OF) and Elevated Plus-Maze (EPM) tests. Firstly, our results showed an increase in exploration behaviors and locomotive functions, together with a decrease of anxiety-linked behaviors in unirradiated *Ptch1*^+/−^ mice compared to WT controls when both Open Field (Figure 4A–C) or Elevated Plus-Maze tests (Figure 4D–G) were used. It is interesting to note that 8 months after irradiation, the behavior of *Ptch1*^+/−^ mice showed an inversion, displaying an increased level of anxiety and decreased activity with respect to their unirradiated controls. In particular, results from the Open Field test showed a significant decrease in the number of rearings in *Ptch1*^+/−^ mice 8 months after irradiation (Figure 4A). Moreover, compared to the unirradiated control, both the irradiated WT and *Ptch1*^+/−^ mice showed a significant decrease in the total distance travelled (Figure 4B–C). Rearing is considered a useful marker of environmental novelty and can facilitate learning about the spatial environment. A decrease in exploration in a novel environment often means that ambulation and rearing are positively correlated [41]. The decrease observed in these parameters suggests an increase in anxiety-like behaviors, also confirmed by data obtained from the Elevated Plus-Maze test. In this test the irradiated *Ptch1*^+/−^ mice showed a significant decrease in activity (total distance travelled and number of head dips, Figure 4D,G) and increased anxiety characterized by open arm avoidance (time and number of entries into the open arms, Figure 4D,F) compared to *Ptch1*^+/−^ unirradiated mice.

Taken together, our data for the Open Field and Elevated Plus-Maze tests show a modulation of cognitive functions by the constitutive activation of the Shh pathway which is manifested as a decrease in locomotion and increase in anxiety levels.

## 3. Discussion

The brain undergoes ionizing radiation exposure in many clinical situations, particularly during radiotherapy for malignant brain tumors. Radiotherapy-related neurocognitive defects are prevalent in children, where they represent a major detrimental side effect of life-saving procedures [42,43] and correlate with the age at treatment, a younger age being associated with more severe neurocognitive deficiencies. 

Accumulating evidence in animal models suggest that radiation-induced cognitive decline involves damage in multiple neural cell types, causing structural and functional alterations in the brain-blood barrier and in glial cell populations, reducing neurogenesis in the hippocampus, altering neuronal function, and increasing neuroinflammation [18,19,44,45]. 

Experimental animal models may shed light on the factors influencing the brain radiation response. In agreement with many published reports, WT mice displayed a progressive impairment of neurogenesis with a significant suppression of all the stem cell populations in the subgranular zone of the DG, and a reduction of new neurons at 2 months post-irradiation. These deficits worsened at 8 months post-irradiation, indicating that neurogenesis is progressively impaired in WT mice in the long term after irradiation with 5 Gy of X-rays. Of note, in WT mice this impairment was associated with exclusive upregulation of genes involved in the injury response such as *adora2A* (*A2AR*) and *ep300*. *ep300* knockdown has been shown to inhibit apoptosis by disrupting the p53-mediated response to DNA damage [46,47], and *A2AR* plays an important role in radiation-induced skin injury, that benefits from its blockade. Moreover, inhibition of A2AR following brain injury is reported to protect oligodendrocytes against apoptosis and to enhance proliferation of their precursors in white matter [48]. Therefore, given the role of these two genes in apoptosis and the protection conferred by their knockdown it is conceivable that their long-term activation might be involved in the deficits of hippocampal neurogenesis observed in WT mice. In addition, *Alk* and *Cxcl1* genes are exclusively downregulated in WT mice and it is noteworthy to mention that loss of function of both these genes is associated with reduced neurogenesis and alteration of behavioral measures, such as activity and anxiety levels [49,50,51], providing a further link between our molecular and phenotypic data in WT mice.

SHH is a key morphogen in the normal development of the CNS of vertebrates, such as the neural tube and brain, which is involved in the control of pattern formation, cell-fate-specification and maintenance of the proliferation, survival and differentiation of neurons in the CNS. In addition to acting as a mitogen for cells with stem-cell-like properties, SHH also promotes the axonal growth of young neurons soon after they are generated from progenitor cells [52,53]. Deregulation of the Shh signal pathway leads to the occurrence of a wide range of neurological diseases, including autism, depression, dementia, stroke, Parkinson’s disease, Huntington’s disease, motor disorders, epilepsy, demyelinating disease, neuropathy and brain tumors [33]. Recently, *Shh* deregulation has emerged as an important modulator of adult hippocampal neurogenesis, oxidation, inflammation, and autophagy [54]. We have previously shown that constitutive activation of the Shh pathway in *Ptch1* heterozygous mice leads to morphological, cellular and molecular alterations in the DG, including elongation and reduced width of the DG, as well as deregulations at multiple steps during lineage progression from neural stem cells to neurons [29]. 

Several recent studies reported the potential neuroprotective therapeutic effects of Shh pathway manipulation in neurodegenerative disorders, cerebral ischemia and other pathologies [55,56,57]. In addition, it was shown that transient activation of the Shh pathway after irradiation rescued salivary gland function by preserving salivary stem/progenitor cells and parasympathetic innervation [58]. However, given that the potential of Shh pathway manipulation in the recovery of radiation injury is still unexplored, we here investigated the in vivo consequences of constitutive activation of the Shh pathway on the hippocampal radiation response. At 2 months post-irradiation the effect of exposure to 5 Gy of X-rays at P10 was milder in *Ptch1*^+/−^ mice compared to WT mice, as they were protected from a deficit in the production of newborn neurons. Moreover, in the *Ptch1*^+/−^ mice the deficits from irradiation of all the cell populations in the subgranular zone were completely recovered after 8 months. Therefore, our results show, for the first time, that the constitutive activation of the Shh pathway in *Ptch1*^+/−^ mice robustly mitigates the radiation-induced inhibition of neurogenesis, by preserving radiosensitive populations of neural stem cells and progenitors, as well as newborn neurons in the DG. Notably, these results suggest an interesting potential role for Shh pathway manipulation among the strategies aimed at reducing radio-induced breakdown in the production of new neurons. Accordingly, our molecular analysis shows that irradiated *Ptch1*^+/−^ mice overexpress several proneural genes, suggesting that combinatorial interactions between transcription factors upon constitutive activation of the Shh pathway, may establish a positive regulatory loop overcoming radiation-induced impairment of neurogenesis. The fibroblast growth factor (*Fgf2*) gene is an SHH-responding proneural gene able to restore hippocampal functions in a model of Alzheimer’s disease, partly as a result of enhanced neurogenesis [59,60]. Moreover *Ptch1*^+/−^ mice overexpress *Nrg1*, a proneural gene able to increase hippocampal proliferation and neurogenesis within the hippocampus when peripherally administered to young adult mice [61]. *Nrg1* overexpression can significantly attenuate neuronal death induced by oxygen-glucose deprivation in primary hippocampal neurons [62] and reduce ROS levels, increasing cell viability [63]. 

In general, our molecular analysis revealed a higher number of deregulated genes in *Ptch1*^+/−^ compared to WT mice with respect to their matched unirradiated controls. These include genes involved in cell differentiation (*Nrg1*, *Pafah1b1*, *Pax5*, *Ptn*), synaptic functions (*Chrm2*, *Fgf2*, *Pafah1b1*, *S100b*, *Apbb1*, *ApoE*) and also transcription factors involved in the regulation of neurogenesis (*Ptn, Flna*). Many of these deregulated genes have implications in cognition, such as *Nrg1*, the overexpression of which, in addition to selectively increasing proliferation and overall neurogenesis, causes behavioral alterations [61,64], or *Chrm2*, which has been implicated in higher cognitive processing [65]. *Pafah1b1* is the gene encoding the protein LIS1, involved in many aspects of neurogenesis, including cell differentiation, synaptic function regulation and neural migration, as well as being associated with lissencephaly, a neuronal migration disorder affecting the cerebral, hippocampal and cerebellar cortices [66,67]. Therefore, all these genes might be potential crucial mediators of the SHH-related regulatory molecular mechanisms providing neuroprotective and anti-inflammatory effects to the stress response in the adult hippocampus.

Experimental evidence shows that *Ptch* zebrafish mutants with constitutive activation of Shh signaling overexpress the *Disrupted-In-Schizophrenia* 1 gene (*Disc1)*, suggesting that *Disc1* functions downstream of SHH in the zebrafish CNS [68]. Notably, three of the genes we found deregulated in the irradiated *Ptch1*^+/−^ hippocampi (*Sox2*, *Pax5*, and *Dll1*), were shown to be mediators of the *Disc1*-driven effects when *Disc1 was* overexpressed or silenced embryonically in mouse hippocampal neural stem precursor cells in vitro. Instead, in irradiated WT mice only *Sox2* was downregulated, allowing us to speculate that the mitigation of radiation-induced effects observed in *Ptch1*^+/−^ mice might be dependent on the molecular interactions between the Shh signaling pathway and *Disc1*, converging on the modulation of *Pax5*, *Sox2* and *Dll1*. 

Our findings also indicated important differences in the hippocampal astrocyte cell population between *Ptch1*^+/−^ and WT mice, with a lower number of astrocytes in *Ptch1*^+/−^ mice, suggesting that constitutive activation of the Shh pathway influences astrocyte homeostasis in the healthy brain. The network of astrocytes constitutes about 50% of the entire glial cell population, with diverse functions, including synaptic transmission modulation and secretion of growth factors [69,70]. In response to CNS injury, astrocytes become activated and respond by hypertrophy of their cellular processes and an increased production of the intermediate glial fibrillary acidic protein (GFAP) [71,72]. These permanent and irreversible modifications, known as reactive astrogliosis, have been described after brain exposure to ionizing radiation [73,74] and can persist from weeks to months to years after injury. Astrogliosis can occur following a single dose [75] or fractionated doses of radiation [76]. Concordantly, our data show that, following an initial depletion, irradiation induces a significant long-term increase of astrocytes in the hippocampus of WT mice. Instead, this was not observed in *Ptch1*^+/−^ mice, indicating that activation of the Shh signaling pathway also protects from radiation-induced active astrogliosis. This was in line with recent findings showing that Shh signaling is active in astrocytes, mitigates inflammation under pathological conditions, and may provide a neuroprotective benefit to the restoration of tissue homeostasis following injury by reducing the permeability of the blood–brain barrier and limiting the extravasation of blood-derived inflammatory molecules into the CNS [77,78]. Moreover, growing evidence indicates that astrocytes participate not only in synaptic and neural circuit functions but also in various behaviors of an organism [79,80].

Noteworthily, astrocytes have a central role in the regulation of adult neurogenesis and changes in molecules produced by astrocytes have been shown to affect either positively or negatively different steps of adult neurogenesis. Pathological conditions, such as inflammation, lead to NSC dysfunction, altering the neuron-astrocyte production rate in favor of astrocytes [81,82], highlighting the importance of a tight control of the stem cell fate. *Fgf2*, exclusively upregulated in irradiated *Ptch1*^+/−^ mice, is mainly produced by astrocytes and it is known to act as a proliferation inducing factor of NSCs [83,84]. Moreover, the FGF2/FGF receptor 1 pathway has been reported to inhibit astrocyte-mediated neuroinflammation in the hippocampus in vitro and in vivo after infrasound exposure [85,86]. Therefore, *Fgf2* upregulation may provide a relevant molecular link between *Shh* hyperactivation and protection of the hippocampus from both radiation-induced neurogenesis impairment and astrogliosis. *Fgf2*, is also known as an extracellular inducer of the orphan nuclear hormone receptor COUP-TFII (chicken ovalbumin upstream promoter transcription factors II) [87]. COUP-TFII is a downstream target of Shh in P19 mouse cells [88]. Furthermore, Shh pathway activation in COUP-TFII mutant cells accounts for the imbalance of excitatory/inhibitory neuron differentiation in the primary somatosensory cortex, leading to the behavioral deficits of neurodevelopmental disorders, including autism spectrum disorders [89]. Therefore, since COUP-TFs are known to control the neuron-astroglia cell fate decision and are key modulators of neuroinflammation in the adult hippocampus [90,91], it is possible to hypothesize that Shh constitutive activation may contribute to mitigate the detrimental radiation effects in the hippocampus of *Ptch1*^+/−^ mice through an *Fgf2*-mediated modulation of COUP-TFs receptors, although further studies are needed to prove it. 

*Ptch1* heterozygosity it is known to alter cognitive behaviors in mice, including reducing motor learning ability and performance on a spatial memory task, therefore linking dysregulation of the Shh signaling pathway with hyperactivity and altered social behaviors [29,92]. Very recently *Ptch1*^+/−^ female mice were shown to have an increased activity compared to WT mice by traveling greater distances in both open-field and partner preference tasks [93]. Social behavior was also reported to be modified sex-specifically in *Ptch1*^+/−^ females that interacted more with both novel and familiar animals in the partner preference task compared to WT controls of the same sex [88]. In agreement with these published data and with our previous indications that Shh-dependent neuroanatomical brain changes, including DG elongation, may alter social behavior in *Ptch1*^+/−^ mice [29], we here show an increase in the exploration behaviors and locomotive functions in unirradiated *Ptch1*^+/−^ mice compared to WT controls, even though our experimental groups included mice of both sexes and not only females. However, it is interesting to note that 8 months after irradiation, the behavior of *Ptch1*^+/−^ mice showed an inversion, displaying an increased level of anxiety and decreased activity with respect to their unirradiated controls. This was not observed in WT mice, where only a decrease in the overall activity (Open Field test) was detected. Although, despite full recovery of the neurogenesis deficit, the persistence of behavioral disturbances in *Ptch1*^+/−^ mice at 8 months post-irradiation was somewhat unexpected, it should be considered that (i) behavioral abnormalities were also observed in unirradiated *Ptch1*^+/−^ mice, (ii) that irradiation was not targeted to the hippocampus but delivered to the whole-body, and (iii) it was carried out at P10, the most vulnerable period for the brain, in which the slightest disturbance may affect the development and result in neurotoxicity in adult mice [94]. Therefore, subtle and complex interactions between the constitutive Shh pathway activation and radiation injuries, also extending to other brain regions besides the hippocampus, may account for the alteration in anxiety and locomotion activities in irradiated *Ptch1*^+/−^ mice. However, further evaluations with a wider range of behavioral tests may help to highlight behavioral dysfunction, demonstrating the role of Shh in functional recovery after radio-induced injury. 

Several studies reveal that manipulation of the Shh pathway carries therapeutic potential in neurodegenerative disorders and cerebral ischemia [56,57,95,96,97,98,99]. After stroke the Shh pathway is upregulated in multiple cell types and post-stroke treatment with an SHH agonist in a distal middle cerebral artery occlusion model leads to functional improvement in the subacute phase after stroke [100]. Treatment with an Shh pathway agonist in stroke mice resulted in enhanced functional recovery in locomotor and cognitive functions, as well as in neurogenesis by increasing the survival of newly born cells derived from both subventricular zone and subgranular zone neural stem cells, total DCX+ neuroblast cells, and neurons (NeuN+/YFP+) in the ischemic brain [101]. It was also reported that treatment with a different Shh pathway agonist (Purmorphamine) administered at an early post-stroke stage (6 h) leads to decreased neuronal damage, BBB integrity and reduced reactive astrogliosis [102]. Finally, anatomical and behavioral deficits in a mouse model of Down syndrome have also reportedly been corrected by a single injection with an Shh signaling pathway agonist (SAG) at birth, suggesting that temporary Shh pathway activation could lead to long-term modulation of brain development and functions [103]. On the other hand, a massive shortfall in PTCH1 and GLI1 was observed in the hippocampus of aged Alzheimer disease transgenic mice that would compromise the ability of genesis in both NSC and glial precursor cells [104]. According to the above evidence, our findings support a role for Shh pathway manipulation to provide the therapeutic possibility of improving brain repair and functional recovery after radiation injury. Future experiments in which irradiated WT mice are treated with an Shh pathway agonist are required to establish a role for Shh pathway manipulation against radiation-induced detrimental consequences in the brain.

## 4. Materials and Methods

### 4.1. Animals and Irradiation

Female and male mice lacking one *Ptch1* allele (*Ptch1*^neo6−7/+^, named *Ptch1*^+/−^ throughout the text), generated through the disruption of exons 6 and 7 in 129/Sv embryonic stem cells and maintained on a CD1 background, were bred and genotyped as described in [28]. Animals were housed under conventional conditions with food and water available ad libitum and a 12-h light cycle. All the experiments were carried out in accordance to the Directive 2010/63/EU for animal experiments. Experimental protocols were reviewed by the Institutional Animal Care and Use Committee, and permission was issued by the “Ministero della Salute” (Approval number is 365/2015-PR).

X-ray irradiation was performed using a Gilardoni CHF 320G X-ray generator (Gilardoni S.p.A.; Mandello del Lario, Lecco, Italy) operated at 250 kVp, 15 mA, with filters of 2.0 mm of Al and 0.5 mm of Cu. *Ptch1*^+/−^ mice and WT littermates of both sexes were whole-body irradiated with 5 Gy of X-rays 10 days after birth (P10) during the peak of the brain growth spurt, a developmental phase characterized by a marked growth in brain size, and recognized as a critical sensitive period for toxic insults [105]. This was performed without anesthesia to avoid further stress in addition to irradiation (Figure S1A). Additional groups were left untreated as controls. Acute mortality before mice weaning was less than 12% of the P10-irradiated mice (Figure S1B). At the end of the experiments, animals were sacrificed by cervical dislocation.

### 4.2. Immunohistochemistry

Brains from unirradiated and irradiated mice (*n* ≥ 4) were collected at different time points, fixed in 10% buffered formalin and embedded in paraffin wax according to standard techniques. Fixed brain sections were immunostained as described in [106] using the following primary antibodies diluted as indicated by the manufacturer: GFAP (Z0334—Dako, Jena, Germany, 1:500), SOX2 (ab97959—Abcam, Cambridge, UK, 1:500), DCX (18723—Abcam, Cambridge, UK, 1:2000), NeuN (MAB377—Millipore, Darmstadt Germany) and IBA1 (019-19741, Wako, Richmond, VA, USA, 1:500). To analyze adult neurogenesis, the brain of each mouse was cut sagittally to the midline and sections were collected starting at around 300 µm from the midline. To standardize the counting area, cell quantification was performed on three nonoverlapping serially collected sections per mouse, one from each hemisphere, representing the rostral/mid-hippocampus. Images for quantification were taken using the imaging software NIS-Elements BR 4.00.05 (Nikon Instruments Europe B.V., Amesterdam, The Netherlands). All experiments were blind analyzed by an independent researcher to the injury. The number of positive cells in each section of the subgranular zone was expressed per mm of the subgranular zone length. NSCs were counted based on criteria including subgranular zone localization, positive labelling and morphology. Immunohistochemical analysis for NeuN was carried out in a rectangular field of 2000 µm^2^ in the supra- and infrapyramidal blade and in the crest area of the DG. Quantitative analysis of astroglial (GFAP^+^) cells were performed in the molecular layer (ML) of the hippocampus and expressed as positive cells/mm^2^. Representative immunostaining images are reported in the Supplementary Material for each condition (Figures S2 and S3). All data were evaluated with GraphPad Prism 6 statistical software (GraphPad Software Inc., San Diego, CA, USA). Statistical significance between groups for most variables was determined using a Student two-tailed *t*-test with or without Welch’s correction. *p*-values < 0.05 were considered to be statistically significant.

### 4.3. RT2 Profiler PCR Array

Brains were collected at 8 months after irradiation and stored in RNAlater solution (1018087—Qiagen, Hilden, Germany) over-night at 4 °C. Then, DGs were manually dissected under a stereomicroscope according to the procedure illustrated in the video at https://www.jove.com/video/1543/dissection-ofhippocampal-dentate-gyrus-from-adult-mouse (accessed on 17 November 2009) [107] and stored at −80 °C in RNAlater until RNA extraction. To quantify gene expression of 84 genes involved in the neurogenic process we used the RT2 Profiler Mouse Neurogenesis PCR Array (PAMM-404Z, SABiosciences, Qiagen, Hilden, Germany). Total RNA (0.5 µg) from the DGs of irradiated and control mice (*n* = 4), was used on a StepOnePlus™ Real-Time PCR System (Applied Biosystems, Life Technologies, Monza, Italy) according to the manufacturer’s instructions. Only Ct values of <35 were included in the calculations. The relative expression of each mRNA was normalized using the equation 2^−∆∆Ct^. For each genotype, three replicates were carried out and a gene was considered to have a significantly altered expression only if displaying a fold-regulation higher/less than 2, with a *p* value ≤ 0.05. Gene expression was related to the mean expression of all five housekeeping genes included in the array.

### 4.4. Cognitive Tests

Behavioral analyses were carried out in control and irradiated *Ptch1*^+/−^ (*n* = 15 at 0 Gy; *n* = 12 at 5 Gy) and WT mice (*n* = 18 at 0 Gy; *n* = 17 at 5 Gy) of mixed genders at 8 months after irradiation.

Open-Field (OF) Test: An Open Field test was used to assess the anxiety-like and activity levels of the animals. The open field consisted of a wooden square (47 cm × 47 cm) surrounded by a dark wall (40 cm). The area of the maze within 15 cm of the wall was considered as peripheral [108,109], while the rest was the central area. At the beginning of the test mice were placed in the center of the arena and allowed to move freely around the maze to explore the environment for 15 min. To remove olfactory cues from the previous animal, the apparatus was cleaned with 70% ethanol after each observation. The video tracking software Ethovision XT© 14 (Noldus Information Technologies, Wageningen, Netherlands), was used to measure the distance travelled over the maze. Additionally, the number of rearings (vertical standing of mice on hind legs) were registered. During the behavioral testing, indirect lighting was used and lighting levels maintained at ≈100 lux [110,111].

Elevated Plus Maze (EPM) Test: The Elevated Plus Maze test was used to assess anxiety-like levels in mice. The apparatus used has two closed arms (25 × 5 × 16 cm) across from each other and perpendicular to two open arms (25 × 5 × 0.5 cm), with a center platform (5 × 5 × 0.5 cm). In the open arms, a small wall (0.5 cm) is used to decrease the number of falls. The entire apparatus is 50 cm above the floor. Mice were transported to the testing room 30 min before the tests. At the start of the session each animal was placed in the central square and allowed to freely explore the environment for 5 min. To remove olfactory cues from the previous animal, the apparatus was cleaned with 70% ethanol after each observation. Performance was recorded by a video camera and data were analyzed with Ethovision XT© version 11.5 (Noldus Information Technologies) video tracking software to measure the time spent in open arms [112,113]. In addition, the number of head dips (downward movements of the head towards the floor) was registered.

Cognitive Statistical Analysis: Data were reported as the mean ± standard error of the mean (SEM). Homogeneity of variances were analyzed using Levene’s test. If variances were homogeneous, ANOVA was used followed by the Tukey post hoc test to evaluate all dose groups simultaneously. If the variances were not homogeneous, the Kruskal–Wallis test was used. Differences between groups were analyzed using the Mann–Whitney U-test. Moreover, the paired *t*-test was used to compare the two different time points tested. The ANOVA test and post hoc analyses adjusted by Bonferroni’s correction were used to analyze the progression for repeated measures of parameters recorded by the Ethovision XT© software (version 11.5). The level of statistical significance for all tests was established at *p* < 0.05. All data were analyzed by means of the statistical package GraphPad Prism 6 statistical software (GraphPad Software Inc., San Diego, CA, USA).

## 5. Conclusions

**Figure 5:**
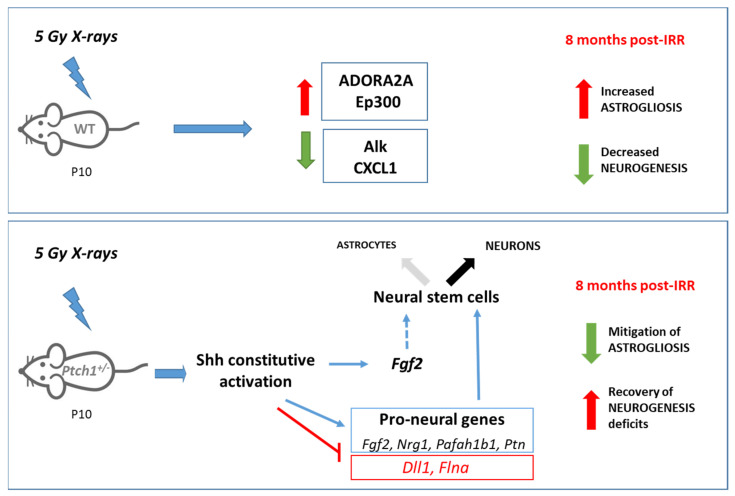
Schematic summary of long-term radiation-induced effects in the hippocampus of WT and *Ptch1*^+/−^ mice. In WT mice, irradiation with 5 Gy of X-rays at P10 induces neurogenesis deficit and astrogliosis, associated with deregulation of genes involved in the injury response (*A2AR* and *ep300* upregulated; *Alk* and *Cxcl1*, downregulated). In *Ptch1*^+/−^ mice we detected a protective role of constitutive Shh pathway activation on neurogenesis deficits and astrogliosis, that was associated with the deregulation of a large number of proneural genes and possibly with a switch from astroglia toward a neuronal cell fate that might contribute to the restoration of neurogenesis.

Originally discovered as a mitogen and crucial for CNS development including patterning and polarity, SHH is now appreciated for its various functions to cope with different types of stresses and multiple potential neuroprotective mechanisms. Of great translational interest, our data, by showing a long-term reduction of the hippocampal deficits in the stem and new neuron compartments and mitigation of astrogliosis induced by P10 irradiation in *Ptch1*^+/−^ mice, demonstrated that constitutive activation of the Shh signaling pathway, by modulating the proneural gene network, protects the hippocampus from radiation damage (Figure 5). This finding has important implications given the prevalence of radiotherapy-related neurocognitive defects in children. However, unexpectedly, deficits in mood and locomotion could still be detected in irradiated *Ptch1*^+/−^ mice, although other brain areas may contribute to these behavioral changes. Our results demonstrated an overall protective effect of constitutive Shh pathway activation on the hippocampal injury derived from perinatal radiation exposure. However, further investigations on the responses triggered by a transient Shh pathway activation to hippocampal recovery following cranial-irradiation, are needed to facilitate its potential clinical application, with particular attention to the establishment of an appropriate time window for intervention and the potential for CNS oncogenic effects.

## Figures and Tables

**Table 1:** Significantly deregulated genes in irradiated mice (WT or *Ptch1*^+/−^ mice) vs. unirradiated CN (WT or *Ptch1*^+/−^ mice) 8 months after irradiation. Upregulated genes (black); downregulated genes (red).

	Synaptic Functions	Cell Differentiation	Apoptosis	Growth Factors	Cell Adhesion/Neural Migration	Cell Cycle/Transcription Factors	Signal Transduction
**WT**	Erbb2	Pax6	Adora2a Ep300 Alk	Cxcl1	Slit2	Ep300	Adora2a Cxcl1
***Ptch1^+/-^***	Chrm2 Fgf2 Pafah1b1 S100b Apbb1 ApoE	Nrg1 Pafah1b1 Pax5	S100b ApoE	Fgf2 Nrg1 Ptn	Pafah1b1 Rac1 Robo1	Ptn Flna	Nrg1 Dll1
**WT** ***Ptch1^+/-^***	App Bdnf Creb1 Dcx Drd2 Map2 Notch1	Bdnf Mdk NeuroD1 Ntf3 Olig2 Sox2	Notch2	Artn Mdk Ndp	Dcx Drd2	Mdk	App Notch1 Notch2

## Data Availability

Other datasets analyzed during the study are available from the corresponding authors on reasonable request.

## Supplementary Materials

Avaiable online at https://www.mdpi.com/article/10.3390/ijms222212605/s1.

## Abbreviations

*Ptch1*	Patched
DG	Dentate Gyrus
Shh	Sonic Hedgehog
SMO	Smoothened
SVZ	Subventricular zone
SGZ	Subgranular zone
GCL	Granular cell layer
WT	Wild Type
CNS	Central nervous system
RGLs	Radial glia-like cells
TAPs	Transit amplifying neural stem cells
DCX	Doublecortin
FC	Fold-changes
GFAP	Glial fibrillary acidic protein
NSC	Neural stem cells
SOX2	Sex determining region Y (SRY) box 2
OF	Open field test
EPM	Elevated plus-maze test
NSPC	Neural stem precursor cell
COUP-TFs	Chicken ovalbumin upstream promoter transcription factors
