# Supplementary material for: Long-Term Effects of Ionizing Radiation on the Hippocampus: Linking Effects of the Sonic Hedgehog Pathway Activation with Radiation Response
Source: Int J Mol Sci. 2021 Nov 22;22(22):12605. doi: 10.3390/ijms222212605 (PMC8624704; doi:10.3390/ijms222212605)
Supplement: Supplementary file 1 [file ijms-22-12605-s001.zip › ijms-1411651-supplementary.pdf]

Supplementary material

Long-term effects of ionizing radiation on the hippocampus: linking the effect of Sonic Hedgehog pathway activation with radiation response

Francesca Antonelli [1*], Arianna Casciati[1], Montserrat Belles[2], Noemi Serra[2], Maria V. Linares-Vidal[2], Carmela Mar ino[1], Mariateresa Mancuso[1], Simonetta Pazzaglia[1*]

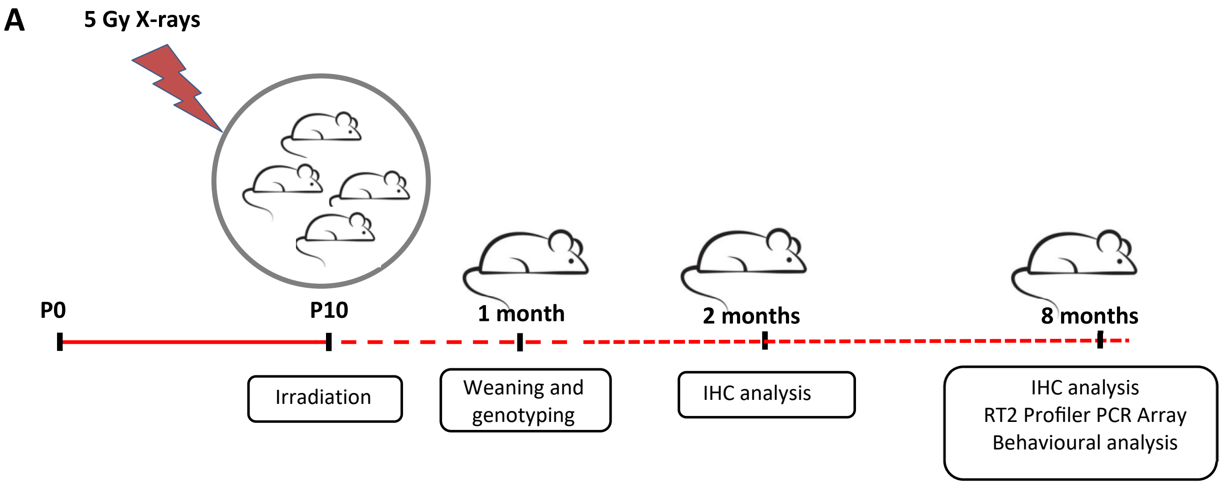

B

WT mice	Sham-irradiated	Irradiated (5 Gy)
2 months	20	9
8 months	42	39 (13)

Ptch$^{+/-}$ mice	Sham-irradiated	Irradiated (5 Gy)
2 months	15	10
8 months	43	42 (22)

Figure S1. (A) Experimental scheme, indicating the time of irradiation and genotyping, as well as the time-points of the different analyses. Irradiation with 5 Gy produced less than 12% of mortality before weaning, when the mouse genotype was still unkown. (B) The table reports the total number of sham- or X-ray-irradiated (5 Gy) mice. In brackets are the number of mice death after weaning, indicating a mortality of 33% for WT mice and 52% for *Ptch1*$^{+/-}$ mice up to 8 months of age.

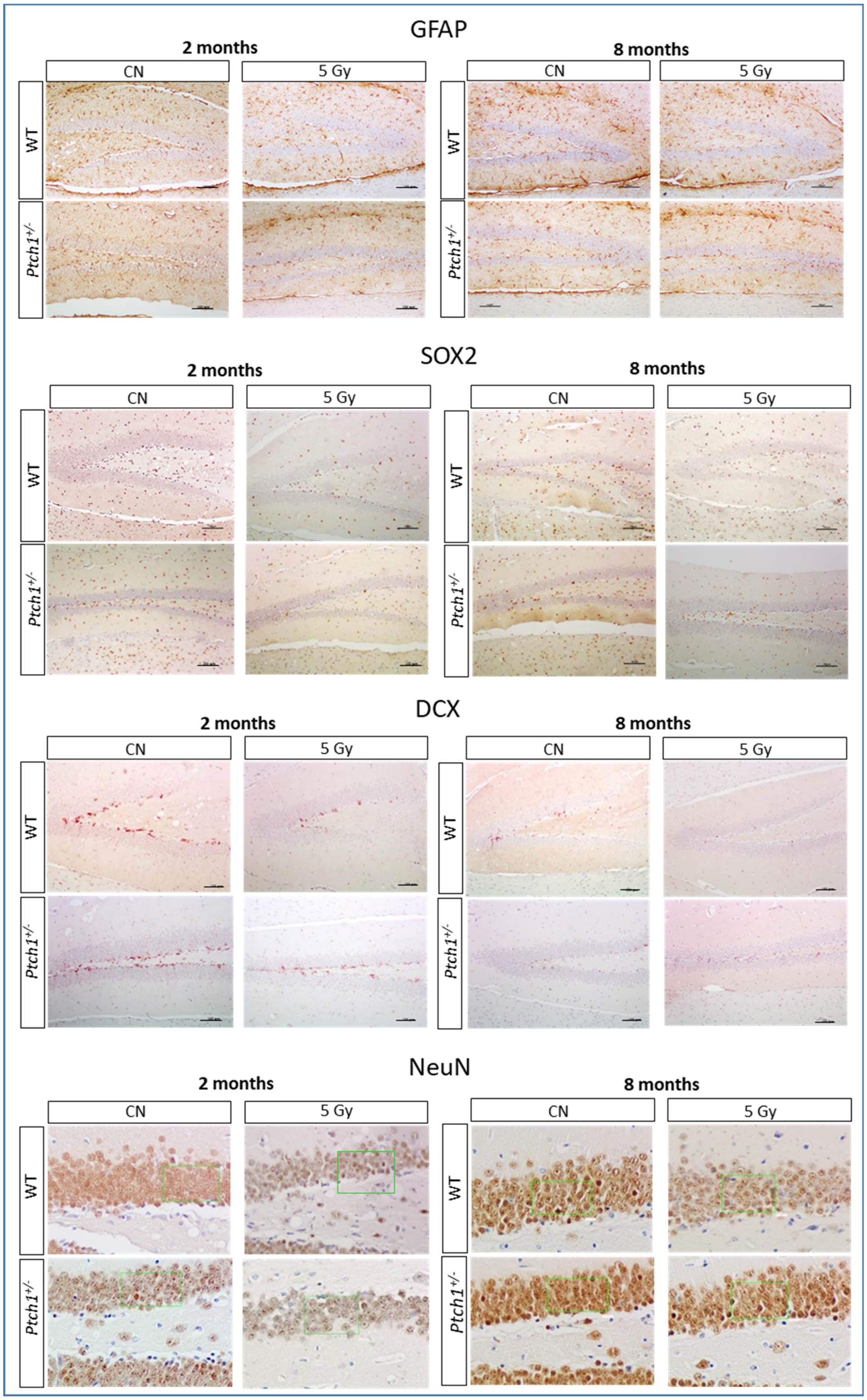

Figure S2. Representative immunostained images for each stage specific neural marker. A) Glial Fibrillary Acidic Protein (GFAP); B) Sex determining region Y (SRY) box 2 (SOX2); (C) doublecortin (DCX) and (D) mature neurons (NeuN). Images, 10X (GFAP, SOX2 and DCX) and 20X (NeuN) magnification, scale bar in the figures. The green rectangular field of 2000 μm^2 in D delineates a representative area in which NeuN analysis was carried out.

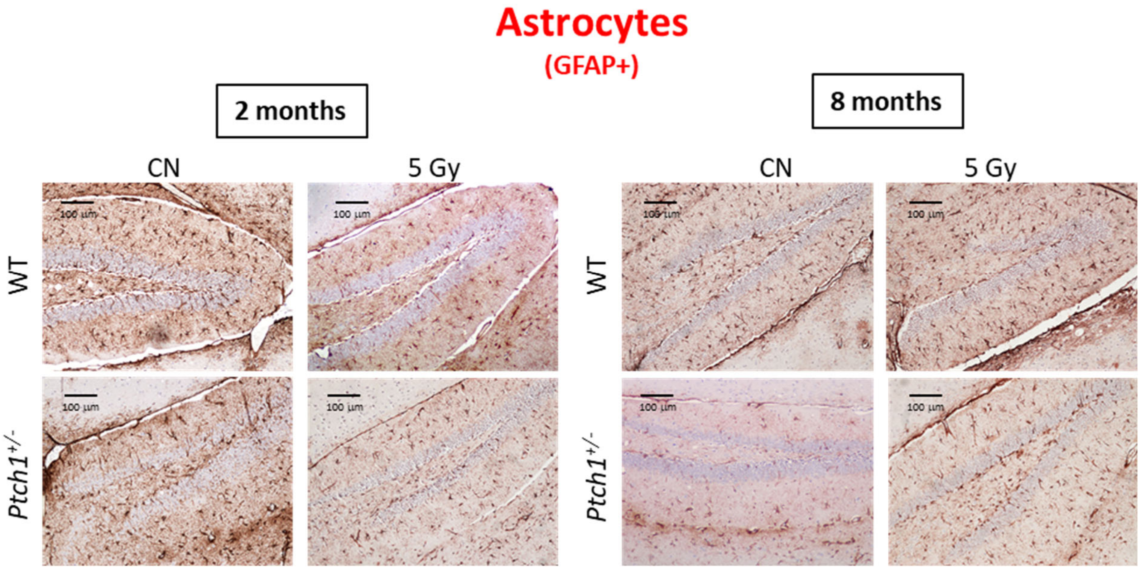

Figure S3. Representative immunostained images for GFAP+ astrocytes. Images, 10X magnification, scale bar in the figures.
